# Selectivity of Dietary Phenolics for Inhibition of Human Monoamine Oxidases A and B

**DOI:** 10.1155/2019/8361858

**Published:** 2019-01-23

**Authors:** Zhenxian Zhang, Hiroki Hamada, Phillip M. Gerk

**Affiliations:** ^1^Virginia Commonwealth University School of Pharmacy, Department of Pharmaceutics, 410 N. 12^th^ Street, Richmond, VA 23298-0533, USA; ^2^Department of Life Science, Okayama University of Science, 1-1 Ridai-cho Kita-ku Okayama, 700-0005, Japan

## Abstract

Monoamine oxidases (MAOs) regulate local levels of neurotransmitters such as dopamine, norepinephrine, and serotonin and thus have been targeted by drugs for the treatment of certain CNS disorders. However, recent studies have shown that these enzymes are upregulated with age in nervous and cardiac tissues and may be involved in degeneration of these tissues, since their metabolic mechanism releases hydrogen peroxide leading to oxidative stress. Thus, targeting these enzymes may be a potential anti-aging strategy. The purpose of this study was to compare the MAO inhibition and selectivity of selected dietary phenolic compounds, using a previously validated assay that would avoid interference from the compounds. Kynuramine metabolism by human recombinant MAO-A and MAO-B leads to formation of 4-hydroxyquinoline, with Vmax values of 10.2±0.2 and 7.35±0.69 nmol/mg/min, respectively, and Km values of 23.1±0.8 *μ*M and 18.0±2.3 *μ*M, respectively. For oral dosing and interactions with the gastrointestinal tract, curcumin, guaiacol, isoeugenol, pterostilbene, resveratrol, and zingerone were tested at their highest expected luminal concentrations from an oral dose. Each of these significantly inhibited both enzymes except for zingerone, which only inhibited MAO-A. The IC50 values were determined, and selectivity indices (MAO-A/MAO-B IC_50_ ratios) were calculated. Resveratrol and isoeugenol were selective for MAO-A, with IC_50_ values of 0.313±0.008 and 3.72±0.20 *μ*M and selectivity indices of 50.5 and 27.4, respectively. Pterostilbene was selective for MAO-B, with IC_50_ of 0.138±0.013 *μ*M and selectivity index of 0.0103. The inhibition of resveratrol (MAO-A) and pterostilbene (MAO-B) was consistent with competitive time-independent mechanisms. Resveratrol 4'-glucoside was the only compound which inhibited MAO-A, but itself, resveratrol 3-glucoside, and pterostilbene 4'-glucoside failed to inhibit MAO-B. Additional studies are needed to establish the effects of these compounds on MAO-A and/or MAO-B in humans.

## 1. Introduction

Monoamine oxidases (MAO) A and B are enzymes found in the mitochondria of the liver and other tissues, metabolizing neurotransmitters such as dopamine, serotonin, and norepinephrine [1]. Several approved therapeutic MAO inhibitors have long been used for the treatment of anxiety and depression and more recently for Parkinson's disease [2]. MAO reaction products include hydrogen peroxide, aldehydes, and ammonia; these may exhibit toxic effects in various tissues. Notably, expression of MAO increases with age by 6-fold for cardiac MAO-A and 4-fold for neuronal MAO-B [3]. As a result, both MAO-A and MAO-B have been investigated for their roles in oxidative stress, aging, and degenerative disease in the heart and the brain [1, 3–6].

In addition to FDA-approved drugs, natural products have also been investigated for their potential MAO inhibition. Some phenolic dietary compounds such as curcumin, eugenol, piperine, quercetin, and resveratrol are not substrates for MAO, but they have inhibitory effects on MAO [7–12]. Curcumin inhibits both MAO-A and MAO-B in mouse brain after p.o. administration [7]. Piperine and paeonol are reversible inhibitors for both MAO-A and MAO-B in rat brain. The mode of inhibition with piperine on MAO-A and MAO-B is mixed and competitive inhibition giving K_i_ values of 35.8 *μ*M and 79.9 *μ*M, respectively [9]. Paeonol has K_i_ values of 51.1 *μ*M and 38.2 *μ*M on MAO-A and MAO-B with non-competitive and competitive inhibition, respectively [9]. Emodin shows mixed mode inhibition on MAO-B with K_i_ values of 15.1 *μ*M in rat brain [9]. Quercetin is a previously established inhibitor of MAO-A [3, 10, 11] and MAO-B [13]. Resveratrol is a potent inhibitor of MAO-A in rat brain with K_i_ of 2.5 *μ*M [12]. Eugenol can competitively inhibit both human recombinant MAO-A and MAO- B with K_i_ of 26 *μ*M and 211 *μ*M [8]. Kaempferol is a selective MAO-A inhibitor [14]; furthermore, certain flavonoid structures have been established as reversible and competitive inhibitors, while glycosidation of these structures decreases or abolishes their MAO inhibition [15]. These phenolic compounds all lack amine groups and therefore MAO inhibition is unexpected and not immediately explained.

The drug-drug interactions between many oral sympathomimetic amines and monoamine oxidase (MAO) inhibitors have been well studied in the literature. The most common adverse effect is high blood pressure. Other adverse effects include headache, chest pain, cardiac arrhythmias, and circulation insufficiency [16]. MAO inhibitors inhibit presystemic and systemic metabolism of some sympathomimetic amines, which are substrates for MAO, resulting in the elevated level of these sympathomimetic amines in circulation [16]. Furthermore, the metabolism of exogenous and endogenous sympathomimetic amines in circulation and tissues could be inhibited by systemic exposure to MAO inhibitors.

Sympathomimetic amines can be divided into two types: direct and indirect acting amines. Indirect acting sympathomimetic amines stimulate the release of noradrenaline from the storage in the sympathetic nerve terminals to interact with postsynaptic adrenergic receptors. MAO inhibitors can increase the level of noradrenaline stored in the nerve terminals. These effects from sympathomimetic amines and MAO inhibitors cause the adverse interaction [16, 17]. Direct acting sympathomimetic amines bind directly to adrenergic receptors. Elimination of these direct acting sympathomimetics from interacting with adrenergic receptors occurs via metabolism by MAO, catechol-O-methyl transferase, and reuptake into presynaptic neurons. Therefore, MAO inhibitors can affect indirectly acting sympathomimetic amines more than directly acting sympathomimetic amines such as phenylephrine [17].

**Figure 1:**
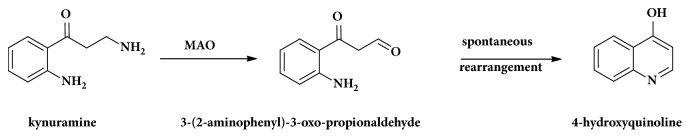
Kynuramine converted to 4-hydroxyquinoline via 3-(2-aminophenyl)-3-oxo-propionaldehyde.

The purpose of this study was to compare the MAO inhibition potential and selectivity of selected phenolic compounds which may have utility in oral dietary supplement products. Since phenolic compounds can act as antioxidants and thus interfere with assay methods depending on the detection of peroxidase activity, a direct chromatographic method measuring the MAO product should yield more reliable results [18]. Therefore kynuramine, a typical substrate of both MAO-A and MAO-B, was used to test if these phenolic compounds can inhibit MAO-A or MAO-B followed by fluorescent HPLC analysis. The metabolite of kynuramine produced by MAO enzymes (3-(2-aminophenyl)-3-oxo-propionaldehyde) rapidly and spontaneously rearranges (by the Schiff base reaction) to the commercially available 4-hydroxyquinoline (shown in Figure 1), which has strong fluorescence for sensitive detection [13].

## 2. Materials and Methods

### 2.1. Chemicals and Reagents

Curcumin (mixture of curcumin, demethoxycurcumin, and bisdemethoxycurcumin) was purchased from Acros Organics (New Jersey, USA). Guaiacol and isoeugenol were purchased from TCI America (Portland, OR). 4-Hydroxyquinoline, zingerone, and trifluoroacetic acid were purchased from Alfa Aesar (Ward Hill, MA). Kynuramine dihydrobromide was purchased from Sigma-Aldrich (St. Louis, MO). Pterostilbene was purchased from ChromaDex (Irvine, CA). Resveratrol was purchased from Beta Pharma, Inc. (New Haven, CT). Piceid (resveratrol 3-glucoside) and vanillin were purchased from TCI America (Portland, OR); *α*-mangostin was purchased from Indofine (Hillsborough Township, NJ); gnetin-C was donated by Hosoda Nutritional (Fukui City, Japan). Silybin was purchased from Cayman Chemicals (Ann Arbor, MI) and chrysin was purchased from Hawkins Pharmaceutical (Roseville, MN). Acetonitrile was purchased from Avantor Performance Materials, Inc. (Center Valley, PA). Dimethyl sulfoxide, perchloric acid (70%), sodium hydroxide, and triethylamine were purchased from Fisher Scientific (Fair Lawn, NJ). Potassium phosphate monobasic was purchased from Sigma (St. Louis, MO). Potassium phosphate dibasic was purchased from J.T. Baker (Phillipsburg, NJ). Recombinant human MAO-A and MAO-B were purchased from BD Biosciences (San Jose, CA) or Corning Discovery Labware (Woburn, MA). Resveratrol 4'-glucoside and pterostilbene 4'-glucoside were synthesized as previously described [19, 20]. All test compounds were dissolved in DMSO.

### 2.2. Chromatographic Conditions and Detection

The chromatographic experiments were conducted by HPLC systems including Waters 2695 separation module, Waters 2487 dual *λ* absorbance detector, and Waters 2475 multi *λ* fluorescence detector (Waters Corporation, Milford, MA). The HPLC method was developed to simultaneously detect and quantify kynuramine and 4-hydroxyquinoline to monitor the enzymatic reaction of recombinant MAO-A/B. The analytical method used was similar with some modifications to those already published and validated [21, 22]. A Microsorb MV C18 column (100 × 4.6 mm, 3 *μ*m, Agilent Technologies) was used at 30°C to separate kynuramine and 4-hydroxyquinoline. The gradient elution was applied at a flow rate of 1 mL/min with 6.5 mM triethylamine and 13 mM trifluoroacetic acid in water as mobile phase A and acetonitrile as mobile phase B, starting at 10% B and increasing to 50%. Kynuramine was detected by UV at 364 nm, and 4-hydroxyquinoline was detected by fluorescence (excitation 316 nm, emission 357 nm). Further details on the HPLC method are in Supplemental Data (available here).

### 2.3. MAO Enzyme Kinetic Assay and K_m_ Determination

The optimized incubation time and human recombinant MAO concentration were selected in the linear range from the time-dependent and MAO concentration-dependent studies (Supplemental Data). Briefly, samples were prepared in potassium phosphate buffer (100 mM, pH 7.4) with a final concentration of MAO in the reaction solution of 0.01 mg/mL, incubated at 37°C for 15 min. Saturation of kynuramine metabolism with MAO-A/B was carried out at concentrations of 2, 5, 10, 25, 50, 100, 250, 500 *μ*M. The enzymatic reactions (200*μ*L) were stopped by 2 N NaOH (75 *μ*L) followed with 70% perchloric acid (25 *μ*L). The samples were vortexed and centrifuged for 5 min at 10,000×g. The supernatant was analyzed by HPLC as described above.

### 2.4. Inhibition Screening and IC_50_ Determination

According to the K_m_ value determined in the experiment described above, the final concentration of kynuramine was set at 10 *μ*M for the inhibition assay, which was less than the K_m_ values for MAO-A and MAO-B. The incubation time was 15 min and MAO concentration was 0.01 mg/mL. The concentration used to screen the inhibitors of MAO-A/B for curcumin, guaiacol, isoeugenol, pterostilbene, resveratrol, and zingerone was 140, 435, 110, 270, 94, and 51 *μ*M, respectively. If the compounds at these concentrations significantly decrease the formation of 4-hydroxyquinoline, further study would be accomplished to determine their IC_50_ for the inhibition of MAO-A/B. Additionally, clorgyline and selegiline were tested as positive controls for MAO-A and MAO-B inhibition [23, 24], while several other natural phenolic compounds were tested for MAO inhibition.

### 2.5. Inhibition Mechanism Studies

To describe the inhibition mechanism for resveratrol or pterostilbene, competition and time dependence were determined. For competition, MAO-A or MAO-B was incubated with varying concentrations of kynuramine (2-450*μ*M) for 30 minutes in the absence (control) or presence of resveratrol (1*μ*M; MAO-A) or pterostilbene (0.45*μ*M). To determine time dependence, the protocol by Obach et al. was followed [25]. Briefly, the enzymes were incubated directly with the inhibitor at 10-fold higher than a quarter of their IC50 values determined herein for resveratrol and pterostilbene for times from 0 to 60 minutes, before diluting 10-fold with kynuramine at a concentration (20*μ*M) approximating its Km values. Tranylcypromine was used as a positive control for time-dependent inhibition [24], and DMSO (0.9% v/v) was used as a negative control [23]. After adding kynuramine, the enzymes were incubated for 30 minutes before processing as described above.

### 2.6. Data Analysis

GraphPad Prism 5 was applied to fit a Michaelis-Menten model to the data to obtain the K_m_ values in the saturation experiments. In the screening experiments, significant differences between control and treated group were determined by a one-way ANOVA followed by Dunnett's posttest (p < 0.05). The condition of the IC_50_ study was incubation of kynuramine (10 *μ*M) and a broad concentration range of inhibitors with MAO-A/B (0.01 mg/mL) for 15 min. GraphPad Prism 5 was applied to fit the data to obtain IC_50_ values by using the concentration-response equation as follows:

(1) $$\begin{array}{c} \frac{v_{i}}{v_{0}}=\frac{1}{1+10^{\wedge }\left(\left(\log\left[I\right]-\log IC_{50}\right)\times \text{Hill Coefficient}\right)} \end{array}$$

This equation included the Hill coefficient as the parameter and could help to characterize the inhibition. If the 95% confidence interval of the Hill coefficient did include 1, the concentration-response equation with the Hill coefficient fixed at 1 was fitted to the data again by the following equation:

(2) $$\begin{array}{c} \frac{v_{i}}{v_{0}}=\frac{1}{1+10^{\wedge }\left(\log\left[I\right]-\log IC_{50}\right)} \end{array}$$

The selectivity index was calculated as a ratio of MAO-B IC_50_/MAO-A IC_50_ for each compound. The calculated solubility values for phenolic dietary compounds (all unionized at pH values ranging from 1 to 7) are from SciFinder [26]. The maximum single doses are from published sources [27–32]. The relevant GI concentration is the lesser of either solubility or the concentration after a single dose.

Time depended data were analyzed by fitting either straight line or one-phase exponential decay models to the data in GraphPad Prism v5.

## 3. Results

### 3.1. Optimized Enzyme Kinetic Assay and K_m_ Determination

**Figure 2:**
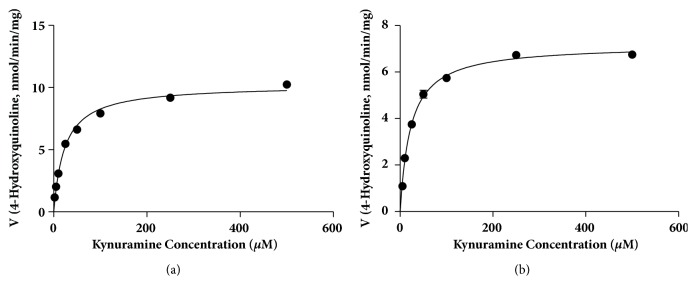
**Concentration dependence of oxidative deamination of kynuramine by MAO-A (a) or MAO-B (b)**. Kynuramine was incubated with MAO-A (a) or MAO-B (b) (0.01mg/ml) for 15 minutes. The formation of 4-hydroxyquinoline (mean ± SD) was determined in three experiments in triplicate; a representative experiment is shown. The Michaelis-Menten model was fitted to the data, represented by the curve.

The concentration dependence for oxidative deamination of kynuramine with MAO-A and MAO-B is shown in Figure 2. Kynuramine (2 to 500 *μ*M) was incubated in 200 *μ*L potassium phosphate buffer (100 mM, pH 7.4) for 15 min with MAO-A or MAO-B (0.01 mg/mL). Michaelis-Menten model was used to fit the data by GraphPad Prism 5. The experiments were conducted 3 times in triplicate. Single representative experiments are shown. For MAO-A, K_m_ and V_max_ were 23.1 ± 0.8 *μ*M and 10.2 ± 0.2 nmol/min/mg (mean ± SEM), respectively. For MAO-B, K_m_ and V_max_ were 18.0 ± 2.3 *μ*M and 7.35 ± 0.69 nmol/min/mg (mean ± SEM), respectively. From these data, the concentration of kynuramine was set at 10 *μ*M for both MAO-A and MAO-B for the following studies so that the kynuramine concentration was < Km.

### 3.2. Inhibition Screening and IC_50_ Determination

**Figure 3:**
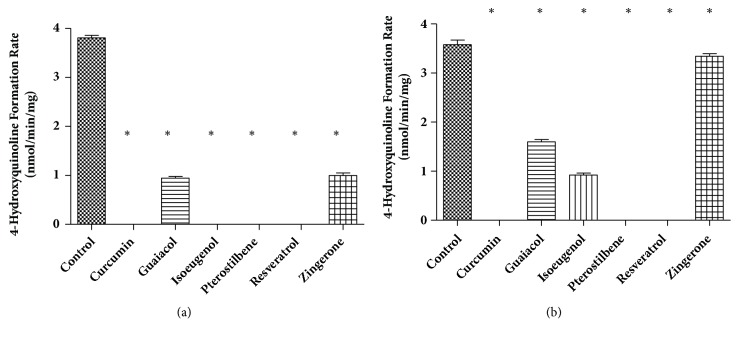
**The inhibition of MAO-A (a) or MAO-B (b) activity by phenolic dietary compounds**. The inhibition screening for oxidative deamination of kynuramine with MAO-A (a) or MAO-B (b) was conducted with kynuramine (10 *µ*M) incubated with either enzyme (0.01 mg/mL) and one of these phenolic dietary compounds. Concentrations tested: curcumin 140*μ*M, guaiacol 435*μ*M, isoeugenol 110*μ*M, pterostilbene 270*μ*M, resveratrol 94*μ*M, and zingerone 51*μ*M. The numbers are expressed as means ± SD; *∗* indicates that the significant differences were analyzed between the control (no inhibitor) and phenolic compounds. Absent bars indicate that the formation of 4-hydroxyquinoline was below the quantification limit.

The inhibition screening for oxidative deamination of kynuramine with MAO-A or MAO-B is shown in Figure 3. Kynuramine (10 *μ*M) was incubated in 200 *μ*L potassium phosphate buffer (100 mM, pH 7.4) for 15 min with MAO-A or MAO-B (0.01 mg/mL) and one of these phenolic dietary compounds. The control was the incubation with kynuramine but without any dietary compounds. The numbers are expressed as means ± SD and the significant differences were analyzed between the control treatment (with no inhibitor) and treatments in presence of phenolic dietary compounds using one-way ANOVA analysis followed by Dunnett's* post hoc *test in GraphPad Prism 5. All the phenolic compounds tested in the experiments showed significant inhibition of MAO-A activity with p < 0.05. All the phenolic compounds tested in the experiments showed significant inhibition of MAO-B activity with p < 0.05. However, zingerone showed less than 10% inhibition at its GI concentration (51 *μ*M) expected from maximum single dose. Therefore zingerone was not investigated further with MAO-B.

**Figure 4:**
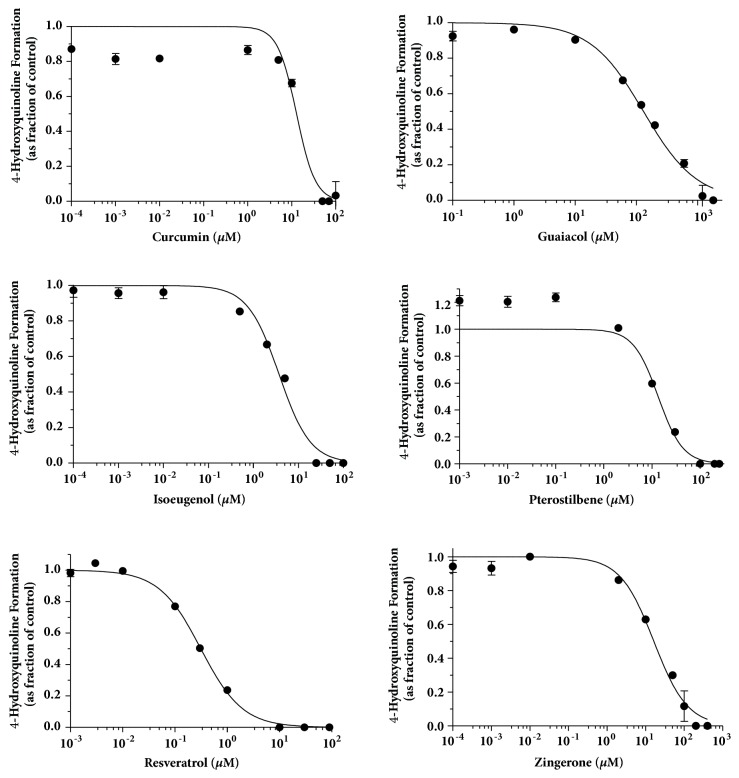
**Determination of IC**_50_ **for curcumin, guaiacol, isoeugenol, pterostilbene, resveratrol, and zingerone on MAO-A activity**. MAO-A activity was measured by the formation of 4-hydroxyquinoline with inhibitors in a broad range of concentrations (at least 10^4^-fold) for 15 min. The Y-axis is expressed as fraction of the control (in absence of inhibitor) and all points on the curves are expressed as means ± SD.

**Figure 5:**
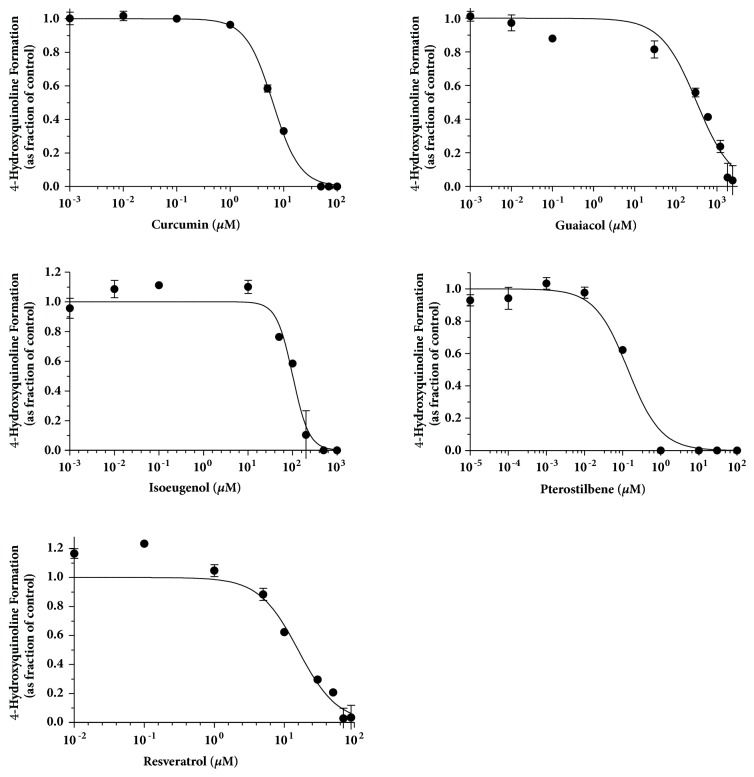
**Determination of IC**_50_ **for curcumin, guaiacol, isoeugenol, pterostilbene, and resveratrol on MAO-B activity**. MAO-B activity was measured by the formation of 4-hydroxyquinoline with inhibitors in a broad range of concentrations (at least 10^4^-fold) for 15 min. The Y axis is expressed as fraction of the control (in absence of inhibitor) and all points on the curves are expressed as means ± SD.

The IC_50_ curves for the inhibitors for kynuramine oxidative deamination with MAO-A are shown in Figures 4 and 5. MAO activities were measured by the formation of 4-hydroxyquinoline with inhibitors in a broad range of concentrations (at least 10^4^-fold) for 15 min incubation of kynuramine with MAO-A or MAO-B. The fractional activity is the value divided by the control (in absence of inhibitor). The formation of 4-hydroxyquinoline was under the lower limit of detection when incubating kynuramine with the negative control for MAO activity. IC_50_ values, Hill coefficients, and selectivity indices are shown in Table 1.

**Figure 6:**
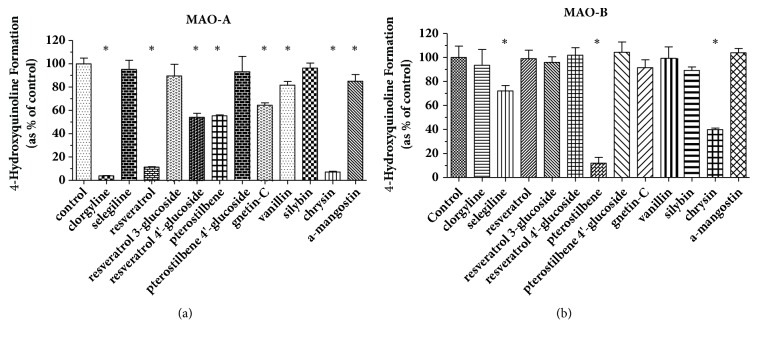
**MAO inhibition by natural phenolic compounds. **Recombinant human monoamine oxidases A (a) and B (b) (0.01mg/ml) were exposed to kynuramine dihydrobromide (10*μ*M) for 15 minutes in the absence (control; n=6) or presence (n=3) of clorgyline (1*μ*M), selegiline (0.5*μ*M), or 15 *μ*M of resveratrol, resveratrol 3-glucoside, resveratrol 4'-glucoside, pterostilbene, pterostilbene 4'-glucoside, gnetin-C, vanillin, silybin, chrysin, or *α*-mangostin. Comparisons were made by one-way ANOVA with Dunnett's posttest; *∗* indicates p<0.05. Control rates for MAO-A and MAO-B were 0.357 ± 0.018 and 0.105 ± 0.010 nmol/min/mg protein, respectively.

In Figure 6, Clorgyline or selegiline selectively inhibited MAO-A or MAO-B as expected [24]. Among the glucosides, only resveratrol 4'-glucoside inhibited MAO-A while MAO-B was not inhibited. As previously reported [23], chrysin inhibited both enzymes.

### 3.3. Inhibition Mechanism

**Figure 7:**
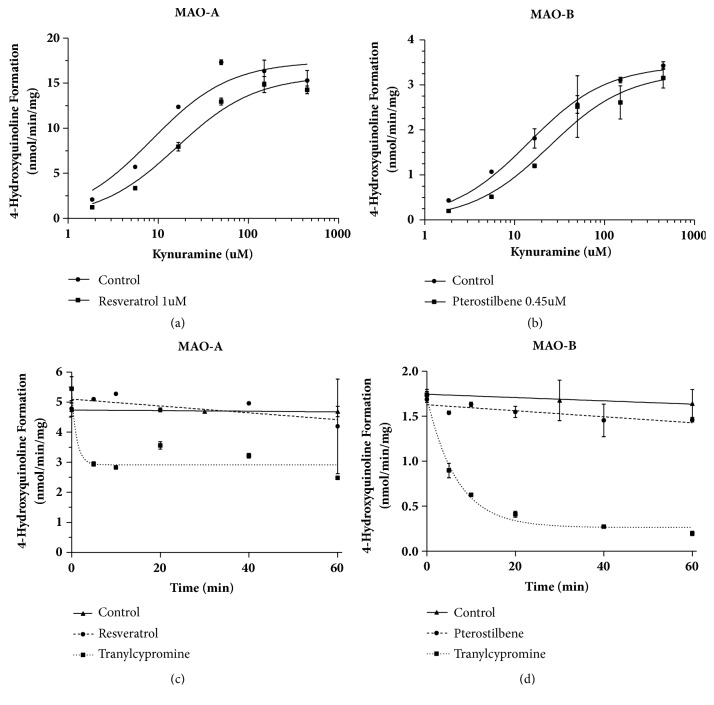
**Mechanism of resveratrol and pterostilbene inhibition of MAO enzymes. **Kynuramine (2-450*μ*M) and MAO-A or MAO-B (0.01mg/ml) were incubated for 15 minutes in the absence or presence of resveratrol (1*μ*M) (a) or pterostilbene (0.45*μ*M) (b). The Michaelis-Menten equation was fit to the data by nonlinear regression to compare any changes in Km or Vmax. (c) MAO-A was preincubated with tranylcypromine (1*μ*M), resveratrol (1.6*μ*M), or DMSO (control) for 0-60 minutes before the addition of kynuramine (20*μ*M). (d) MAO-A was preincubated with tranylcypromine (0.5*μ*M), pterostilbene (0.7*μ*M), or DMSO (control) for 0-60 minutes before the addition of kynuramine (20*μ*M). All samples were incubated at 37°C for 30 minutes. One-phase decay or straight line equations were fit to the data.

Figure 7 showed that in the absence or presence of resveratrol (1 *μ*M), the Vmax and Km values for MAO-A were 17.4 ± 0.7 or 15.8 ± 0.4 nmol/min/mg protein and 8.46 ± 1.56 or 16.1 ± 1.8 *μ*M, respectively. For MAO-B, in the absence or presence of pterostilbene (0.45 *μ*M), the Vmax and Km values were 3.44 ± 0.07 or 3.28 ± 0.19 nmol/min/mg protein and 14.4 ± 1.2 or 24.4 ± 5.4 *μ*M, respectively. MAO-A activity showed dependence upon preincubation time with tranylcypromine (1 *μ*M), with a preincubation half-life of 1.1 minutes, and a plateau showing 48% inhibition. However, resveratrol (1.6*μ*M) preincubation revealed a time profile not significantly different from the control. MAO-B preincubation with tranylcypromine (0.5*μ*M) also showed time dependence, with a half-life of 4.7 minutes, and a plateau at 85% inhibition, while preincubation with pterostilbene (0.7*μ*M) revealed a time profile not significantly different from the control.

## 4. Discussion

Previously validated HPLC methods [21, 23] were adapted to simultaneously quantitate kynuramine and the product of its metabolism by MAO 4-hydroxyquinoline. We applied this assay to facilitate the quantitation and comparison of several phenolic compounds on human MAO enzymes. Interestingly, some of these compounds showed considerable selectivity toward MAO-A or MAO-B. A previous study shows that resveratrol had similar potency for inhibiting MAO-A and MAO-B [33]; however, our study showed that resveratrol had higher potency and selectivity for MAO-A. The reason for the difference is unknown, but may be due to differences in assay methods, i.e., Amplex Red vs. kynuramine. The interference with peroxidase-based assays has been previously established [18].

From the optimization studies (Supplementary Materials), the formation of 4-hydroxyquinoline was linear over 60 min with the protein concentration range of 0.003 mg/mL–0.03 mg/mL, which was comparable with the results from the paper published by Herraiz et al. in 2006 [21]. The K_m_ values of kynuramine oxidative deamination by MAO-A and MAO-B were 23 *μ*M and 18 *μ*M, respectively, which indicated MAO-A has similar affinity toward kynuramine, compared to MAO-B. In the literature, the K_m_ values of kynuramine for human MAO-A and MAO-B were reported as 42 *μ*M and 26 *μ*M [34]. Another study obtained the K_m_ values of MAO-A and MAO-B with kynuramine as 44.1 and 90.0 *μ*M, respectively [35]. K_m_ values reported here were similar to the values in literature, although differences in methods or recombinant enzyme sources may account for differences in reported K_m_ or Vmax values. The concentration of kynuramine for the inhibition study with phenolic compounds was set at 10 *μ*M, which was below the K_m_ value for both MAO-A and MAO-B.

Nonlinear regression revealed Hill coefficients of unity for guaiacol and zingerone showing 1-to-1 apparent binding to MAO-A, and guaiacol and pterostilbene followed 1-to-1 apparent binding to MAO-B. The Hill coefficient of resveratrol with MAO-A was 1.08, while curcumin, isoeugenol, and pterostilbene had Hill coefficients larger than 1, suggesting positive cooperativity, multiple active sites, or non-ideal inhibition behavior [36]. Positive cooperativity could be a possible reason. The binding of the inhibitor to one active site on the enzyme may increase the binding affinity of the inhibitor to other active sites [36]. Another possibility is that the complete inhibition of an enzyme can be achieved by binding of more than one molecule of inhibitor to the enzyme [36]. Further study is required to investigate the mechanism of inhibition which leads to the Hill coefficient larger than 1, including possible allosterism.

Among these tested phenolic dietary compounds, the inhibitory effects on MAO-A and MAO-B in animal models were reported in the literature previously [7, 12]. However, in this study, human recombinant MAO-A and MAO-B enzymes were used as models to test these phenolic compounds. Curcumin can inhibit MAO-A and MAO-B in mouse brain after p.o. administration [7]. We also found curcumin inhibited both MAO-A and MAO-B with IC_50_ values of 12.89 *μ*M and 6.30 *μ*M, respectively. In this study, resveratrol was the most potent inhibitor for MAO-A with IC_50_ as 0.31 *μ*M; its inhibition was consistent with a competitive mechanism, as previously demonstrated by Ryu et al. [12]. Resveratrol is a potent competitive inhibitor of MAO-A in rat brain with IC_50_ of 2 *μ*M and K_i_ of 2.5 *μ*M [12]. Resveratrol was also previously reported to be an inhibitor of MAO-A but did not significantly inhibit MAO-B up to 10*μ*M [18], which is consistent with our study in which resveratrol showed a relatively high IC50 value for MAO-B of 15.8*μ*M (Table 1). Pterostilbene has never been reported as an MAO inhibitor, but it was the most potent MAO-B inhibitor in our study, with an IC_50_ of 0.138 *μ*M.

Compared to the GI concentration converted from the maximum single dose, the IC_50_ values of all phenolic inhibitors on MAO-A and MAO-B are smaller than the maximum concentration in GI tract. The most potent inhibitor for MAO-A was resveratrol followed by isoeugenol, curcumin, pterostilbene, zingerone, and guaiacol in descending order of potency. The most potent inhibitor for MAO-B was pterostilbene followed by curcumin, resveratrol, isoeugenol, and guaiacol in descending order of potency.

Additionally, the selectivity indices expressed as the ratios of MAO-B/MAO-A IC_50_ values (Table 1) showed that resveratrol and isoeugenol are selective MAO-A inhibitors, while pterostilbene is a selective MAO-B inhibitor. These data are especially intriguing, considering that only two methyl groups differentiate resveratrol and pterostilbene. Besides resveratrol, isoeugenol showed the next highest level of potency and selectivity for MAO-A. Isoeugenol has a methylated catechol moiety (as do zingerone and guaiacol) and also a hydrophobic side chain, which may contribute to its MAO-A inhibition exceeding those of zingerone and guaiacol. Furthermore, among the glucosides of resveratrol and pterostilbene tested herein, MAO-A was inhibited only by resveratrol 4'-glucoside, while MAO-B was not inhibited. This is consistent with the loss of inhibitory activity by glycosides of kaempferol [15].

Phenolic compounds are substrates for neither MAO-A or MAO-B and, unlike other MAO, substrates and inhibitors are devoid of any nitrogen atoms. The mechanism of the inhibition of phenolic compounds on MAO is not clear, but none of them was reported to have irreversible inhibition on MAO-A or MAO-B [8, 9]. The researchers found that they are reversible inhibitors with various mode of inhibition such as competitive inhibition, non-competitive, or mixed-type inhibition [8, 9]. Furthermore, several flavonoids have been established as reversible and competitive inhibitors [15].

Published studies show that these phenolic MAO-A inhibitors all have low oral bioavailability in animal models, although this has not been determined in humans. Curcumin has poor bioavailability after oral administration in humans even after a high dose of 12 g/day, which leads to low plasma concentrations [37]. At doses of 4 g, 6 g, and 8 g, the maximum concentration of curcumin in plasma is 0.51 *μ*M, 0.64 *μ*M, and 1.77 *μ*M, respectively [38]. After gavage administration, the absolute bioavailability of isoeugenol in female and male rats is 19% and 10%, respectively. The low bioavailability of isoeugenol was also observed in mice as 28% for male mice and 31% for female mice after gavage bolus [39]. The peak plasma concentration of resveratrol in humans is very low after oral dosing [40, 41]. At 25 mg, 50 mg, 100 mg, and 150 mg doses, the maximum plasma concentrations of resveratrol are 1.48 ng/mL, 6.59 ng/mL, 21.4 ng/mL, and 24.8 ng/mL, respectively [40]. At higher doses of 0.5 g, 1.0 g, 2.5 g, and 5.0 g, the corresponding peak plasma concentrations of resveratrol are 72.6 ng/mL, 117.0 ng/mL, 268.0 ng/mL, and 538.8 ng/mL [41]. The oral bioavailability in rats was determined as 12.5% after 10 mg/kg gavage administration by Lin et al. [42]. After giving rats 56 or 168 mg/kg/day for pterostilbene by gavage for 14 continuous days, the oral bioavailability is 80% [43]. The reason why these phenolic compounds have such low bioavailability is that they all undergo extensive presystemic metabolism and are converted to their metabolites before going to the systemic circulation [41, 43–51].

As described above, in the dose range of 25 mg to 5.0 g, and considering the plasma protein binding of 91% for resveratrol [52], the unbound maximal peak plasma concentrations range from 0.584 to 212 nM. With the IC_50_ values of 0.313 and 15.8 *μ*M for MAO-A and MAO-B and assuming competitive inhibition of resveratrol on human MAO-A and MAO-B, the values of K_i_ would be 0.218 and 10.2 *μ*M for MAO-A and MAO-B, respectively. The drug-drug interaction index (unbound C_max_/K_i_ [53]) for MAO-A is in the range of 2.7 × 10^−3^ to 0.97. Thus at high resveratrol doses (5.0 g), inhibition of MAO-A may occur. However, the drug-drug interaction index for MAO-B is in the range of 5.7 × 10^−5^ to 0.021, making clinically relevant MAO-B interactions with resveratrol unlikely. As with many other natural phenolic compounds, human plasma concentration data and absolute oral bioavailability for oral doses of pterostilbene are lacking [54], making predictions of gastrointestinal or systemic MAO-B interactions speculative. A human clinical study with pterostilbene shows that 125mg twice daily orally for 6-8 weeks reduces both systolic and diastolic blood pressure, although it slightly raised plasma low-density lipoproteins [55]. Another clinical study with pterostilbene 50mg in combination with nicotinamide riboside 250mg for two months also reduced diastolic blood pressure [56]. Acute, severe, systemic MAO inhibition is expected to increase blood pressure and result in serotonin syndrome [2]; however, such adverse reactions were not reported in these studies. As with many dietary compounds, the absolute oral bioavailability of pterostilbene has not been reported, but animal studies show that it is much greater than that of resveratrol [43]. Additional studies are required to determine the absolute oral bioavailability of pterostilbene and other potentially beneficial phenolic compounds and to establish their antiaging effects on human cardiac and neuronal tissues.

Since these phenolic compounds all have relatively low bioavailability, the inhibition occurring after first-pass metabolism is likely to be limited. Most inhibitory effects on MAO-A and MAO-B would be limited to GI tract and liver. Nishimura et al. established the mRNA levels of MAO-A and MAO-B in various human tissues [57]. From their data, the MAO-A mRNA/MAO-B mRNA ratio in the liver is 0.727, whereas the ratio in the small intestine is 4.41, suggesting that MAO-A and MAO-B expression may be similar in the liver, but MAO-A predominates in the intestine. Thus, any effects of MAO-A inhibition in the intestine could be concerning. When other dietary compounds are substrates of MAO and their presystemic metabolism is blocked resulting in higher systemic exposure, MAO inhibition may be problematic. One example is tyramine. Interactions between food constituents and drugs are complicated with various conditions and difficult to predict. When patients take irreversible MAO inhibitors, tyramine-rich foods are to be avoided.

Other assays for MAO activity are available, including peroxidase- and luciferase-based assays. However, antioxidant phenolics can interfere with peroxidase-based assays by quenching the hydrogen peroxide formed [58]. In our lab, resveratrol strongly quenched luminesce in the MAO-glo assay (Promega, Madison, WI; Tamoor Hassan, unpublished data). The metabolic production of 4-hydroxyquinoline could avoid these interferences, but only for compounds which do not interfere with its detection. Thus, the kynuramine-based assay with chromatographic separation offers a solution to avoid interferences. Assays for kynuramine and 4-hydroxyquinoline have been previously reported, and Herraiz et al. showed that antioxidants can indeed interfere with Amplex Red assays, while this interference is avoided by HPLC analysis of 4-hydroxyquinoline formation [18]. The quantitative analysis could be simply achieved by fluorometric assay [59]. The phenolic compounds tested in this study have very strong fluorescence, which may interfere with the fluorescent signal from 4-hydroxyquinoline if measured in a microplate reader. Therefore, a fluorometric microplate assay may not be selective for the detection of 4-hydroxyquinoline and thus chromatographic separation of 4-hydroxyquinoline and the phenolic compounds are required. Other analyses are accomplished by HPLC with UV and fluorescence detection as well as LC-MS/MS [21, 34, 60, 61]. Since 4-hydroxyquinoline has very good fluorescence and kynuramine can be detected by UV detection, HPLC methods with UV and fluorescence detectors are found to be quite adequate for analysis in* in vitro* enzyme kinetic studies. In order to avoid analytical interferences, Herraiz et al. developed a reversed-phase HPLC method by gradient elution with 50 mM ammonium phosphate buffer at pH 3 and 20% of this buffer in acetonitrile [21, 60]. Also, in their HPLC method for 4-hydroxyquinoline, Parikh et al. used a mobile phase containing 0.2mM perchloric acid [23]. In order to avoid the potential for damage to our HPLC system, we modified the mobile phase as discussed in the method section.

The HPLC method for quantitative analysis of kynuramine and 4-hydroxyquinoline used 6.5 mM triethylamine and 13 mM trifluoroacetic acid in water as its aqueous phase, which has a pH value around 2. The estimated most basic pKa value for kynuramine is 8.4, making it cationic in the mobile phase [26]. The estimated most acidic and most basic pKa values for 4-hydroxyquinoline are 4.3 and 11.1, respectively [26]. Hence 4-hydroxyquinoline is also cationic in the mobile phase. At high concentration, trifluoroacetic acid can act as an ion-pairing agent for cations, which can improve kynuramine and 4-hydroxyquinoline retention. When using the aqueous mobile phase with only TFA at 0.05%, there was a tailing problem with the peak shape. This can be caused by the ions like sodium and potassium bound to silanol exchanging with ionized basic analytes at low pH. As an additive in the mobile phase, triethylamine can fix the tailing problem on the column. Excess triethylamine in the mobile phase can replace the ions instead of basic analytes. Therefore, triethylamine can reduce the peak tailing [62].

## 5. Conclusions

In conclusion, we applied a previously validated kynuramine-based MAO activity assay with HPLC separation and fluorescence detection for determining the inhibition and selectivity of several phenolic compounds. Among the compounds tested, resveratrol was potent and selective for MAO-A inhibition, while pterostilbene was potent and selective for MAO-B inhibition. Both compounds appeared to be competitive, time-independent inhibitors. Our calculations suggest that high doses of resveratrol have the potential to inhibit MAO-A in the gastrointestinal tract. Human pharmacokinetic studies with oral dosing of pterostilbene will facilitate future predictions of its clinical potential to interact with MAO-B in the gastrointestinal tract, liver, or systemic circulation. The anti-aging potential of these compounds is worth further investigations.

## Figures and Tables

**Table 1:** Selectivity of phenolic compounds for MAO-A and MAO-B.

	**MAO-A**		**MAO-B**		**Selectivity Index**
	IC_50_ (*µ*M)	Hill coefficient	IC_50_ (*µ*M)	Hill coefficient	
curcumin	12.9 ± 1.3	2.0 ± 0.4	6.30 ± 0.11	1.7 ± 0.1	0.488
guaiacol	131 ± 6	1.0	322 ± 27	1.0	2.46
isoeugenol	3.72 ± 0.20	1.2 ± 0.1	102 ± 5	2.4 ± 0.3	27.4
pterostilbene	13.4 ± 1.5	1.7 ± 0.3	0.138 ± 0.013	1.0	0.0103
resveratrol	0.313 ± 0.008	1.1 ± 0.0	15.8 ± 1.3	1.6 ± 0.2	50.5
zingerone	16.3 ± 1.1	1.0			

## Data Availability

The data used to support the findings of this study are available from the corresponding author upon request.
