# Supplementary material for: Selectivity of Dietary Phenolics for Inhibition of Human Monoamine Oxidases A and B
Source: Biomed Res Int. 2019 Jan 23;2019:8361858. doi: 10.1155/2019/8361858 (PMC6364133; doi:10.1155/2019/8361858)
Supplement: Supplementary Material — s are available describing the HPLC method and the optimization of the enzyme incubation conditions. [file 8361858.f1.pdf]

Supplementary Material

SELECTIVITY OF DIETARY PHENOLICS FOR INHIBITION OF HUMAN MONOAMINE OXIDASES A AND B

Zhenxian Zhang[1], Hiroki Hamada[2], and Phillip M. Gerk[1]*

[1]Virginia Commonwealth University School of Pharmacy, Department of Pharmaceutics

410 N. 12th Street, Richmond, VA23298-0533, USA

[2]Department of Life Science, Okayama University of Science

1-1 Ridai-cho Kita-ku Okayama, 700-0005, JAPAN

*Corresponding Author: pmgerk@vcu.edu

A. HPLC Methods and Revalidation

The HPLC method was previously developed [1] to simultaneously detect and quantify kynuramine and 4-hydroxyquinoline to monitor the enzymatic reaction of recombinant MAO-A/B. A Microsorb MV C18 column (100 × 4.6 mm, 3 µm, Agilent Technologies) was used at 30°C to separate kynuramine and 4-hydroxyquinoline. The gradient elution was applied at a flow rate of 1 mL/min with 6.5 mM triethylamine and 13 mM trifluoroacetic acid in water as mobile phase A and acetonitrile as mobile phase B (shown in **Table S1**). Kynuramine was detected by UV at 364 nm, and 4-hydroxyquinoline was detected by fluorescence (excitation 316 nm, emission 357 nm).

Table S1. The Gradient Elution for Kynuramine and 4-Hydroxyquinoline

Time (min)	Mobile Phase A	Mobile Phase B
0	90	10
1	90	10
5	50	50
7	90	10
8	90	10

Previously, HPLC assays for MAO-mediated metabolism of kynuramine to 4-hydroxyquinoline have been used to examine inhibition of MAO enzymes [2, 3]. In our inhibition studies with phenolic dietary compounds, the HPLC method had to be modified to separate the phenolic compounds from kynuramine and 4-hydroxyquinoline to avoid interference. When doing inhibition studies with guaiacol, the gradient method was modified as follows: 10% B in A was maintained for 1 min and then mobile phase B was increased to 50% in 4 min. After kynuramine and 4-hydroxyquinoline were eluted successfully, mobile phase B was decreased to the original 10% in 2 min and maintained for 5 min. When doing inhibition studies with isoeugenol, pterostilbene, and zingerone, the gradient method was modified as follows: 10% B in A was maintained for 1 min and then mobile phase B was increased to 50% in 4 min.

The mobile phase B was further increased to 90% in 2 min and maintained for 2 min. After all the compounds eluted successfully, the mobile phase B was decreased to the original 10% in 2 min and maintained for 4 min. These modifications did not change the retention time and the peak shape of kynuramine and 4-hydroxyquinoline, but helped the elution of the phenolic dietary compounds after kynuramine and 4-hydroxyquinoline to avoid interference in the following runs. The extension in run time was not expected to affect validation parameters.

Stock Solution Preparation

The reference standards of kynuramine and 4-hydroxyquinoline were dissolved in DMSO to obtain stock solution with a concentration of 50 mM and 200 mM, respectively. Further stock solutions were prepared by diluting the stock solution with DMSO. All the stock solutions were stored at -80 ^{o}C and protected from the light.

Preparation of Standard Curves and Quality Controls

The matrix solution was made by mixing MAO (0.01 mg/mL) in potassium phosphate buffer (100 mM, pH 7.4), 2 N NaOH, and 70% perchloric acid in the ratio of 8:3:1. Standard curves were prepared freshly by spiking the stock solutions in the prepared matrix solution with a concentration range of 2 – 1000 μM for kynuramine and 0.050 – 30 μM for 4-hydroxyquinoline, which covered the concentrations in the samples.

The quality controls were prepared freshly by spiking the stock solutions in the prepared matrix solution with LLOQ (2.00 μM for kynuramine and 0.050 μM for 4-hydroxyquinoline), low quality control (10.0 μM for kynuramine and 0.25 μM for 4-hydroxyquinoline), medium quality control (300 μM for kynuramine and 7.5 μM for 4-hydroxyquinoline), high quality control (600 μM for kynuramine and 15 μM for 4-hydroxyquinoline).

Sample Preparation

For standards curves and quality controls, samples were vortexed and centrifuged for 5 min at 10,000 ×g at room temperature. The supernatant was taken and transferred to an autosampler vial. The volume injected into the HPLC was 100 μL. For enzymatic samples, 2 N NaOH (75 μL) was added into the reaction mixture and followed by 70% perchloric acid (25 μL) to stop the reaction as well as precipitate the protein. Then the samples were vortexed and centrifuged for 5 min at 10,000 ×g at room temperature. The supernatant was taken and transferred to an autosampler vial. The volume injected into the HPLC was 100 μL.

Method Validation

The linearity of standard curve was determined by GraphPad Prism 5 using the model line through origin or first-order polynomial (straight line). The r^2 values from fitting were required to be larger than 0.99. The lower limit of quantitation (LLOQ) was determined with the criterion that the signal to noise ratio was 10:1 when compared to blank samples from matrix. For determination of intra-assay accuracy and precision, the quality control samples at LLOQ, low, medium, and high concentrations were assayed six times within the same run. For determination of inter-assay accuracy and precision, the quality control samples at LLOQ, low, medium, and high concentrations were assayed six times in three separate runs. The recoveries were determined as follows: the quality control samples at LLOQ, low, medium, and high concentrations were compared with the samples spiked at the same final concentrations after sample preparation. For sample processing stability, the quality control samples at LLOQ, low,

medium, and high concentrations were prepared and kept in the autosampler at 4 °C for 40 hrs and then injected into the HPLC for analysis. The criterion for stability was the detected concentrations of the quality control samples should be less than 15% change of the nominal spiked concentrations for low, medium, and high concentrations. For LLOQ, the detected concentration of the quality control sample should be less than 20% change of the nominal spiked concentrations.

The standard curves for kynuramine were linear from 2 to 1000 μM with $r^2 > 0.99$. The standard curves for 4-hydroxyquinoline were linear from 0.050 to 30 μM with $r^2 > 0.99$. The LLOQ for kynuramine and 4-hydroxyquinoline were 2.00 μM and 0.050 μM, respectively. The intra-assay accuracy and precision for kynuramine and 4-hydroxyquinoline are listed in **Tables S2** and **Table S3**. The DFN and RSD for LLOQ were within 20%. The bias and RSD for other quality control concentrations were within 15%.

Table S2.The Intra-assay Accuracy and Precision for Kynuramine

Kynuramine Concentration (μM)	N	Mean	DFN	RSD
2.00	6	2.01	0.6%	4.1%
10.0	6	10.0	0.0%	0.2%
300	6	304	1.4%	0.2%
600	6	605	0.9%	0.1%

Table S3.The Intra-assay Accuracy and Precision for 4-Hydroxyquinoline

4-Hydroxyquinoline Concentration (μM)	N	Mean	DFN	RSD
0.050	6	0.058	15.9%	5.0%
0.25	6	0.25	1.1%	0.2%
7.5	6	7.3	-2.1%	0.3%
15	6	14	-3.8%	0.2%

The inter-assay accuracy and precision for kynuramine and 4-hydroxyquinoline are listed in **Tables S4** and **S5**. The bias and RSD for LLOQ were within 20%. The bias and RSD for other quality control concentrations were within 15%.

Table S4.The Inter-assay Accuracy and Precision for Kynuramine

Kynuramine Concentration (μM)	N	Day 1	Day 2	Day 3	Mean	DFN	RSD
2.00	6	2.01	2.01	2.00	2.01	0.5%	0.2%
10.0	6	10.0	10.0	10.0	10.0	-0.1%	0.3%
300	6	304	304	305	305	1.5%	0.2%
600	6	605	601	606	604	0.7%	0.5%

Table S5. The Inter-assay Accuracy and Precision for 4-Hydroxyquinoline

4-Hydroxyquinoline Concentration (µM)	N	Day 1	Day 2	Day 3	Mean	DFN	RSD
0.050	6	0.058	0.058	0.060	0.059	17.2%	1.8%
0.25	6	0.25	0.25	0.26	0.25	1.5%	1.6%
7.5	6	7.3	7.3	7.5	7.4	-1.6%	1.5%
15	6	14	14	15	15	-3.5%	1.9%

The average recoveries for kynuramine at LLOQ, low, medium, and high concentrations were 98.0%, 99.1%, 100.8%, and 100.2%, respectively. The average recoveries for 4-hydroxyquinoline at LLOQ, low, medium, and high concentrations were 101.8%, 99.8%, 101.1%, and 100.2%, respectively.

The stability tests for kynuramine and 4-hydroxyquinoline in the autosampler at 4 ^{o}C for 40 hrs were listed in **Table S6** and **Table S7**. The bias and RSD for LLOQ were within 20%. The bias and RSD for other quality control concentrations were within 15%.

Table S6. Sample Processing Stability for Kynuramine

Kynuramine Concentration (µM)	N	Mean	DFN	RSD
2.00	6	1.96	-1.8%	4.5%
10.0	6	10.0	0.1%	0.2%
300	6	305	1.7%	0.1%
600	6	607	1.1%	0.1%

Table S7. Sample Processing Stability for 4-Hydroxyquinoline

4-Hydroxyquinoline Concentration (µM)	N	Mean	DFN	RSD
0.050	6	0.059	18.3%	5.2%
0.25	6	0.26	2.2%	0.4%
7.5	6	7.4	-0.9%	0.4%
15	6	15	-2.5%	0.2%

B. Optimization of Enzyme Incubations

Time-dependent and MAO concentration-dependent studies were conducted to optimize the enzyme kinetic assay for kynuramine with MAO-A and MAO-B. Briefly, kynuramine (11.11 µM) in 180 µL potassium phosphate buffer (100 mM, pH 7.4) was made from the stock solution in DMSO and pre-warmed to 37°C for 5 min before initiation of the enzymatic reaction. The DMSO concentration in the final reaction buffer was < 0.5%. After pre-incubation, MAO-A/B (0.1 mg/mL) in 20 µL potassium phosphate buffer (100 mM, pH 7.4) was added and mixed with the kynuramine solution to initiate the reaction. The final concentration of kynuramine and MAO-A/B was 10 µM and 0.01 mg/mL in 200 µL reaction solution for the time-dependent study. The enzymatic reaction was stopped by 2 N NaOH (75 µL) followed with 70% perchloric acid (25 µL) at incubation times of 10, 20, 30, 40, and 60 min. The samples were vortexed and centrifuged for 5 min at 10000 × g. The supernatant was taken and injected to the HPLC with the method discussed above. For assessment of protein concentration-dependence, the same concentration of kynuramine (11.11 µM) was prepared in 180 µL potassium phosphate buffer

(100 mM, pH 7.4) and pre-warmed for 5 min. Various MAO concentrations (0.003, 0.01, 0.03, 0.1, 0.3 mg/mL) in 20 μL potassium phosphate buffer (100 mM, pH 7.4) were added and the total protein concentration was kept constant at 0.3 mg/mL by standardizing with the MAO control. The incubation time was 15 min, which was selected based on the results from the time-dependent study that are discussed below in the result section.

The time-dependent study for oxidative deamination of kynuramine with MAO-A is shown in **Figure S1**. Kynuramine (10 μM) was incubated with MAO-A (0.01 mg/mL) in 200 μL potassium phosphate buffer (100 mM, pH 7.4) for 10, 20, 30, 40, and 60 min. The formation of 4-hydroxyquinoline was analyzed after the enzymatic reaction. A simple linear model without y-intercept was used to fit the data with GraphPad Prism 5. The formation of 4-hydroxyquinoline was linear over 60 min with the rate of 3.28 ± 0.09 nmol/mg/min (mean ± SEM) and $r^2 = 0.9887$. According to the results from this study, the incubation time was selected as 15 min for the following enzymatic assay.

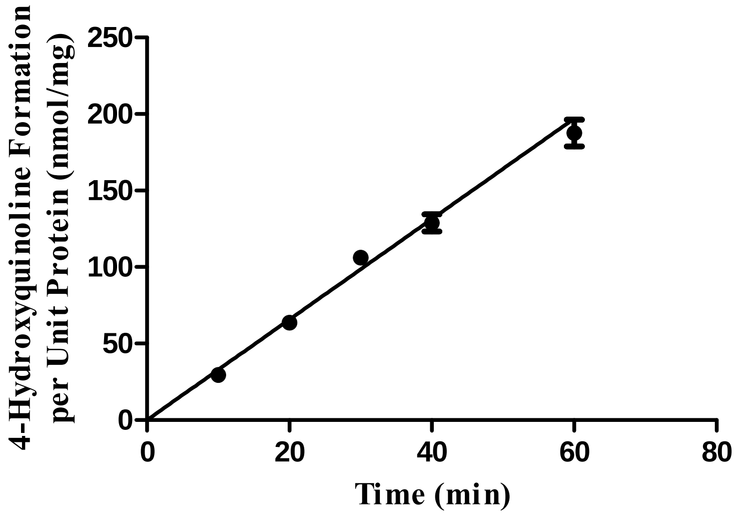

Figure S1. Time-dependence for Oxidative Deamination of Kynuramine with MAO-A. Kynuramine (10 μM) was incubated with MAO-A (0.01 mg/mL) for 10, 20, 30, 40, and 60 min. Line through origin model was used to fit the data with GraphPad Prism 5. The formation of 4-hydroxyquinoline per unit protein (mean ± SD) was linear over 60 min with the rate of 3.28 ± 0.09 nmol/mg/min (mean ± SEM) and $r^2 = 0.9887$.

The time-dependent study for oxidative deamination of kynuramine with MAO-B is shown in **Figure S2**. Kynuramine (10 μM) was incubated with MAO-B (0.01 mg/mL) in 200 μL potassium phosphate buffer (100 mM, pH 7.4) for 10, 20, 30, 40, and 60 min. The formation of 4-hydroxyquinoline was analyzed after the enzymatic reaction. A simple linear model without y-intercept was used to fit the data with GraphPad Prism 5. The formation of 4-hydroxyquinoline was linear over 60 min with the rate of 2.70 ± 0.07 nmol/mg/min (mean ± SEM) and $r^2 = 0.9841$. According to the results from this study, the incubation time was selected as 15 min for the following enzymatic assay.

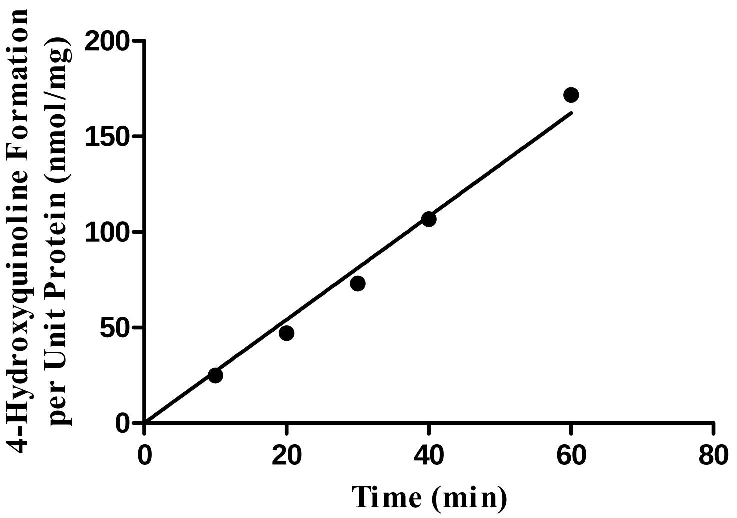

Figure S2. Time-dependence for Oxidative Deamination of Kynuramine with MAO-B. Kynuramine (10 μM) was incubated with MAO-B (0.01 mg/mL) for 10, 20, 30, 40, and 60 min. Line through origin model was used to fit the data with GraphPad Prism 5. The formation of 4-hydroxyquinoline per unit protein (mean ± SD) was linear over 60 min with the rate of 2.70 ± 0.07 nmol/mg/min (mean ± SEM) and $r^2 = 0.9841$.

The MAO concentration-dependent study for oxidative deamination of kynuramine with MAO-A is shown in **Figure S3**. Kynuramine (10 μM) was incubated with MAO-A (0.003, 0.01, 0.03 mg/mL) in 200 μL potassium phosphate buffer (100 mM, pH 7.4) for 15 min. The total protein concentration was kept constant at 0.03 mg/mL by compensating with MAO control (BD Biosciences). The formation of 4-hydroxyquinoline was analyzed after the enzymatic reaction and showed linearity over 0.03 mg/mL MAO-A with the rate of of 3.06 ± 0.03 nmol/mg/min (mean ± SEM) and $r^2 = 0.9970$. According to the results from this study, the MAO-A concentration was selected as 0.01 mg/mL for the following enzymatic assay.

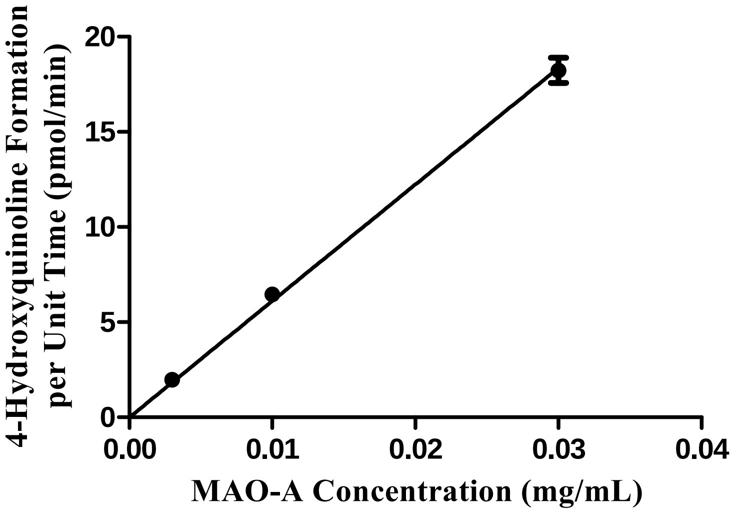

Figure S3. MAO Concentration-dependence for Oxidative Deamination of Kynuramine with MAO-A. Kynuramine (10 μM) was incubated with various concentrations of MAO-A (0.003, 0.01, 0.03 mg/mL). Line through origin model was used to fit the data with GraphPad Prism 5. The formation of 4-hydroxyquinoline per unit time (mean ± SD) was linear over 0.03 mg/mL MAO-A with the rate of 3.06 ± 0.03 nmol/mg/min (mean ± SEM) and $r^2 = 0.9970$.

The MAO concentration-dependent study for oxidative deamination of kynuramine with MAO-B is shown in **Figure S4**. Kynuramine (10 μM) was incubated with MAO-B (0.003, 0.01, 0.03 mg/mL) in 200 μL potassium phosphate buffer (100 mM, pH 7.4) for 15 min. The total protein concentration was kept constant at 0.03 mg/mL by compensating with MAO control (BD Biosciences). The formation of 4-hydroxyquinoline was analyzed after the enzymatic reaction and showed linearity over 0.03 mg/mL MAO-B with the rate of of 3.66 ± 0.08 nmol/mg/min (mean ± SEM) and $r^2 = 0.9942$. According to the results from this study, the MAO-B concentration was selected as 0.01 mg/mL for the following enzymatic assay.

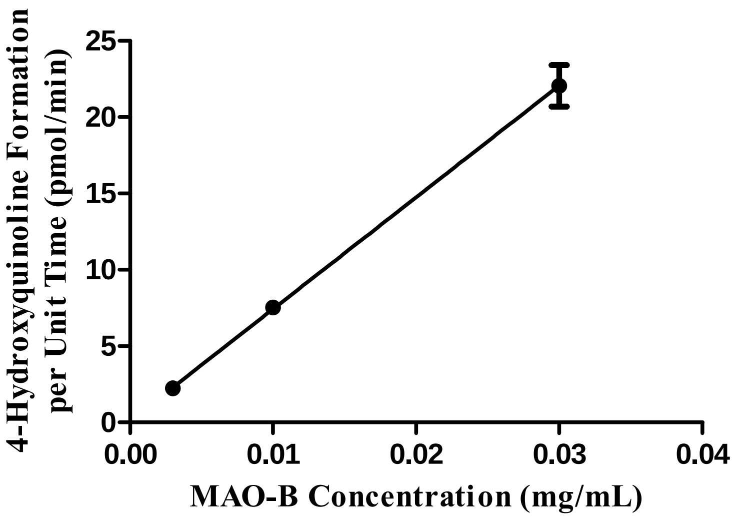

Figure S4. MAO Concentration-dependence for Oxidative Deamination of Kynuramine with MAO-B. Kynuramine (10 μM) was incubated with various concentrations of MAO-B (0.003, 0.01, 0.03 mg/mL). First order polynomial (straight line) model was used to fit the data with GraphPad Prism 5. The formation of 4-hydroxyquinoline per unit time (mean ± SD) was linear over 0.03 mg/mL MAO-B with the rate of 3.66 ± 0.08 nmol/mg/min (mean ± SEM) and $r^2 = 0.9942$.

Reference

1. Parikh S, Hanscom S, Gagne P, Crespi C and Patten C. A fluorescent-based, high-throughput assay for detecting inhibitors of human monoamine oxidase A and B. *Drug Metab Rev* 2002; 34: 164
2. Herraiz T and Chaparro C. Analysis of monoamine oxidase enzymatic activity by reversed-phase high performance liquid chromatography and inhibition by beta-carboline alkaloids occurring in foods and plants. *Journal of chromatography* 2006; 1120: 237-43
3. Yan Z, Caldwell GW, Zhao B and Reitz AB. A high-throughput monoamine oxidase inhibition assay using liquid chromatography with tandem mass spectrometry. *Rapid Communications in Mass Spectrometry* 2004; 18: 834-840. DOI: doi:10.1002/rcm.1415
